# Kausalität, Evidenz und Subjektivität: Paul Martinis Methodenkritik der Psychosomatischen Medizin

**DOI:** 10.1007/s00048-021-00316-5

**Published:** 2021-11-04

**Authors:** Hans-Georg Hofer

**Affiliations:** Institut für Ethik, Geschichte und Theorie der Medizin, WWU Münster, Münster, Deutschland

**Keywords:** Klinische Medizin, Innere Medizin, Epistemologie, Evidenz, Kausalität, Paul Martini, Clinical Medicine, Internal Medicine, Epistemology, Evidence, Causality, Paul Martini

## Abstract

Der Internistenkongress von 1949 war Anlass und Arena einer breit wahrgenommenen Kontroverse zu epistemologischen Fragen der Psychosomatischen Medizin. Der Beitrag verortet zunächst den Kongress in der Nachkriegsgeschichte und zeichnet die Verlaufslinien der Debatte nach. Dabei werden sowohl die Positionen der Sprecher der Psychosomatischen Medizin – Viktor von Weizsäcker und Alexander Mitscherlich – als auch diejenige von Paul Martini, der auf Basis seiner Methodologie klinischer Forschung grundsätzliche Kritik äußerte, rekonstruiert. In einem zweiten Schritt werden die jeweils unterschiedlichen Auffassungen von Kausalität, Evidenz und Subjektivität herausgearbeitet und kontextualisiert. Das Hauptaugenmerk liegt hierbei auf dem konkreten und expliziten Gebrauch dieser Begriffe bei Martini. Schließlich zeige ich, dass diese Ereignisse im Jahr 1949 als Kulmination einer fortdauernden Kontroverse über wissenschaftliche Evidenz in der klinischen Medizin gelesen werden können, die sich mit ihren Beteiligten und Bezugsebenen über mehrere Jahrzehnte erstreckte.

## Abstract (en)

The 1949 congress of internal medicine saw a heated and widely perceived controversy on epistemological issues of psychosomatic medicine. This article begins by outlining the place and significance of the congress in post-war history and tracing the course of the debate. The positions of the proponents of psychosomatic medicine, Viktor von Weizsäcker and Alexander Mitscherlich, are reconstructed, as well as those of the internist Paul Martini, who offered fundamental criticisms on the basis of his methodology of clinical research. In a second step, the respective different understandings of causality, evidence, and subjectivity are elaborated and contextualized. A special focus is on Martini’s explicit use of these terms as well as his further research initiatives. Finally, I argue that “1949” can be analyzed as the culmination of an ongoing controversy about scientific evidence in clinical medicine that spanned several decades with its participants and levels of reference.

## 1949 Revisited: Der Wiesbadener Internistenkongress und seine Kontroversen

Der Internistenkongress des Jahres 1949 hatte ein evidentes Momentum: Im Gründungsjahr der Bundesrepublik an seinen angestammten Ort in Wiesbaden zurückgekehrt, stand er für Tradition und Aufbruch zugleich. Als Vorsitzender firmierte Curt Oehme (1883–1963), Leiter der internistischen Poliklinik in Heidelberg. Der Kongress wurde das erhoffte Großereignis. Etwa 2.500 Ärzte, darunter auch zahlreiche internationale Teilnehmer, waren nach Wiesbaden gekommen. Für Fred Mielke (1922–1959), Alexander Mitscherlichs (1908–1932) Mitarbeiter und Mitverfasser der Dokumentensammlung des Nürnberger Ärzteprozesses, war es die „größte wissenschaftliche Tagung der Nachkriegszeit“ (Mielke 1949). Ein anonymer Tagungsteilnehmer nannte in der englischen Zeitschrift *The Lancet* den Kongress „an imposing affair“ und berichtete mit trockenem Humor von einer sportlich-robusten Kongressorganisation, von „nonstop performances“ enthusiastischer Vortragender – und von verzweifelten Sektionsleitern, die nicht nur ihre Glocke, sondern sogar ihren Körper einsetzen mussten („physical intervention“), um Vortragende zum Abschluss ihres jeweiligen Vortrags bewegen zu können.^1^

Die erste Zusammenkunft der Internisten nach dem Krieg war es jedoch nicht. Die Deutsche Gesellschaft für Innere Medizin hatte ein Jahr zuvor, 1948, ihre jährlichen Kongresse bereits wiederaufgenommen. Die Kongresseinrichtungen in Wiesbaden waren allerdings so stark zerstört, dass die Zusammenkunft einmalig in Karlsruhe abgehalten wurde – unter dem Vorsitz des Bonner Internisten Paul Martini (1889–1964). Ihm war die Rolle des Reorganisators der deutschen Inneren Medizin zugefallen (Martini 1948a; Forsbach & Hofer 2017). Wenngleich Oehme satzungsgemäß das Amt des Vorsitzenden von ihm übernahm, hatte Martini maßgeblich Einfluss auf den „eigentlichen“ Kongress im April 1949 in Wiesbaden, einschließlich seiner fachlichen Positionierung und seinen Schwerpunktsetzungen. Er drängte auf die Wiederaufnahme internationaler Kontakte, beriet Oehme in Fragen des Umgangs mit den Militärregierungen und machte seinerseits Vorschläge für mögliche Vortragende aus etlichen europäischen Staaten sowie aus den USA und Kanada. Als Vertrauter von Konrad Adenauer (1876–1967) informierte er zudem über Entwicklungen der „westdeutschen Zentralregierung“.^2^

Der Kongress von 1949 stand für eine Rückkehr zur Normalität und verband Traditionspflege und Zukunftsgewandtheit. Er zeigte jedoch auch eine Tendenz zu Selbstvergessenheit und einer selektiven Erinnerungspolitik. Erstmals seit 1938 wurden wieder Ehrenmitgliedschaften verliehen. Mit Gustav von Bergmann (1878–1955) und Alfred Schittenhelm (1874–1954) wurden ausgerechnet jene Internisten zu Ehrenmitgliedern ernannt, die sich dem Nationalsozialismus angedient sowie an der Selbstgleichschaltung und Entlassungspolitik ihrer Fakultäten mitgewirkt hatten (von Bergmann als Prorektor der Charité, Schittenhelm in Kiel).^3^ Verstörend und aus heutiger Sicht kaum nachvollziehbar war insbesondere die Verleihung der Ehrenmitgliedschaft an Schittenhelm. Dieser hatte 1933 in der Fachgesellschaft die Absetzung des gewählten „jüdischen“ Vorsitzenden Leopold Lichtwitz (1876–1943) vorangetrieben, danach selbst den Vorsitz übernommen und eine rigorose Anpassungspolitik an das NS-Regime durchgesetzt. Zur *hidden agenda* des Kongresses gehörte weiterhin die vom Vorstand der Fachgesellschaft betriebene Initiative, für die im Nürnberger Ärzteprozess verurteilten Mitglieder der Fachgesellschaft – darunter Wilhelm Beiglböck (1905–1963) und Siegfried Handloser (1885–1954) – Unterstützung in Form entlastender Gutachten und Petitionen zu organisieren. Auch in der Frage der Vergangenheitspolitik setzte der Kongress somit Maßstäbe (Forsbach & Hofer 2018: 164).

Im Selbstverständnis war die deutsche Medizin zurück auf der Bühne, das Comeback geglückt. Der Frankfurter Klinikdirektor Franz Volhard sprach davon, dass der Internistenkongress „schon immer das Antlitz der wissenschaftlichen Medizin“ geformt habe und nun, „im Zeitalter der modernen Therapie“, den nächsten Meilenstein setze: „Die gemeinsame medizinische Forschung der ganzen Welt ist ein gutes Stück vorwärtsgekommen“ (Volhard 1949). Das war etwas großspurig, aber nicht unzutreffend gesagt. Der Kongress hatte eine beeindruckende thematische Spannweite und war eine Leistungsschau der Zusammenarbeit von grundlagenbezogener, naturwissenschaftlicher und klinisch-therapeutischer Forschung. Er verhandelte Chemotherapie, Conteben, Penicillin, Streptomycin, Zytostatika (Hartmann 1949). Wiesbaden 1949 war nicht zuletzt ein Kommunikationsforum „therapeutischer Revolutionen“ – und ließe sich in diese Richtung noch eingehender untersuchen (Greene et al. 2016).

Im Gedächtnis blieb der Kongress vor allem durch die leidenschaftlich ausgetragenen Kontroversen um die Psychosomatische Medizin.^4^ Von ihren Proponenten in einen Gegensatz zur „naturwissenschaftlichen Medizin“ gebracht, wurden hier grundsätzliche methodische und epistemologische Auffassungsunterschiede sichtbar, die über den Kongress hinaus zur anhaltenden Reflexion und Positionierung herausforderten.^5^

### Psychosomatische Medizin: Weizsäcker und Mitscherlich

Die Psychosomatische Medizin hatte Curt Oehme als Kongressvorsitzender prominent platziert; sie war das erste Verhandlungsthema. Als Vortragende waren Viktor von Weizsäcker (1886–1957) und Alexander Mitscherlich bestimmt worden, die als Tandem zur Psychosomatischem Medizin sprachen. Die Vorträge fanden zu einem Zeitpunkt statt, wo Mitscherlich um eine eigene psychosomatische Abteilung an der Heidelberger Klinik rang – und Weizsäcker nach Möglichkeiten suchte, sein Anliegen der anthropologischen Medizin erneut zu positionieren – „jetzt einig mit künstlerischen, politischen, philosophischen Suchbewegungen, welche der schweren Geburt einer neuen Weltepoche vorangehen“, wie er im zeitlichen Umfeld des Kongresses schrieb (Weizsäcker 1949a: 6).

Weizsäcker begann mit einem anthropologisch relativierten Wissenschaftsverständnis. Wissenschaft sei „eine ganz bestimmte menschliche Haltung unter anderen“, Forschung eine „Umgangsform“; „wissenschaftliche Objektivität nicht sakrosankt“ und sogar potenziell gefährlich, da sie „Subjektivität vernichtet“ (Weizsäcker 1949b: 15). Psychosomatische Medizin anthropologischer Provenienz und Prägung sei nicht ohne Abkehr möglich, und zwar von der „naturwissenschaftlichen Medizin“ und vom „modernen Betriebsstaat“. Dabei erinnerte er an Freud, dessen Psychoanalyse er von der „reichsdeutschen Psychiatrie misshandelt“ sah – und wandelte eine in den Ohren des Auditoriums vertraut klingende Parole ab: „Die psychosomatische Medizin muß eine tiefenpsychologische sein oder sie wird nicht sein“ (Weizsäcker 1949b: 16; in Anlehnung an Naunyn 1905: 349).

Inhaltlich waren Weizsäckers Forderungen weder neu noch überraschend. Er forderte die Einführung des Subjekts in die Medizin. Psychosomatische Medizin war aus seiner Sicht Beziehungsmedizin: „Zelle verkehrt auch mit Zelle, Organ mit Organ, Lebewesen mit Lebewesen, Seele mit Seele, mit sich selbst und anderen“. In diesem Zusammenhang fällt der Satz: „Körper und Seele sind keine Einheit, aber sie gehen miteinander um“ (Weizsäcker 1949b: 17). Daraus ergaben sich für Weizsäcker erkenntnistheoretische, programmatische und praktische Konsequenzen: (1) Den Kausalitätsbegriff lehnte er strikt ab: „Seelisches drückt sich in der Körpersprache aus, Körperliches in der seelischen; das ist keine Kausalität“ (Weizsäcker 1949b: 20). Was sich gemeinsam ausdrückt, ist Weizsäcker zufolge mit „Sinn“ hinterlegt, ist „Organsprache“, die entziffert werden will. (2) Arzt und Patienten stehen in ihrem Denken, Handeln und Fühlen in einem unauflöslichen Verhältnis; sie sind beide Subjekte. Ihre Subjektivität ist intrinsisch, nicht reduzierbar und damit (3) eine notwendige Bedingung für die Gestaltung der therapeutischen Beziehung mit dem Patienten: „Therapie, das heißt: ärztliches Handeln, beteiligt sich am Krankheitsvorgang, begleitet ihn, vermischt sich mit ihm, wirkt mit am Verlauf.“ Therapie war solcherart nicht Intervention, sondern Teilhabe, Voraussetzung und Vehikel zur „menschlichen Vervollkommnung“ (Weizsäcker 1949b: 22; Geisthövel 2016).

Insgesamt war dies ein kämpferischer Auftritt im Wortkleid des Revolutionären. Weizsäcker suchte die Zuspitzung und zog die Auseinandersetzung ins Grundsätzliche. Die Koexistenz von Altem und Neuem lehnte er ab, denn beides könne nicht an ein- und demselben Ort sein: „Die recht verstandene psychosomatische Medizin hat einen umstürzenden Charakter“ (Weizsäcker 1949b: 21). Felix Anschütz (1920–2014), der internistischen Psychosomatik nahestehend, erinnerte einen „politisch aggressiven Vortrag“ und eine emotionale Auseinandersetzung mit Paul Martini (Anschütz 1990: 19). Von jener „staunenden Trauer“, die Mitscherlich einige Jahre später an Weizsäcker beobachtet haben wollte (Mitscherlich 1956), keine Spur.

Mitscherlichs anschließender Vortrag hatte eine ähnliche Stoßrichtung, doch waren subtile Differenzen erkennbar. Mitscherlich arbeitete Weizsäcker nicht nach und auch nicht zu, sah sich nicht als Adlatus der Anthropologischen Medizin. Er konzedierte zunächst, um verbindliche Signale bemüht, dass aus der Inneren Medizin selbst wichtige Anregungen gekommen seien. Zugleich nahm er auf US-amerikanische Autoren der *Psychosomatic Medicine* (u. a. Franz Alexander, Flanders Dunbar) Bezug, um „Anschluss an den Stand der Forschung in der Welt [zu] gewinnen“ (Miterschlich 1949: 38).

Psychosomatische Medizin war Mitscherlich zufolge durch eine Umkehr des medizinischen Aufgabenkreises charakterisiert. Anders als Weizsäcker, der jeden wesensmäßigen Vorrang im Umgang von Körper und Seele ablehnte, priorisierte er die „Beeinflussungsrichtung“ vom Psychischen zum Somatischen, wollte dies jedoch ebenfalls nicht als Kausalbeziehung gedeutet wissen. Aufgabe der Psychosomatischen Medizin sei es, „von der manifesten Krankheit zurück zum Sinn des Geschehens zu fragen.“ An diesem Punkt klappte Mitscherlich das Visier auf und widersprach coram publico Paul Martini:

Wir dürfen uns nicht die Tatsache verschleiern, daß hier *grundsätzliche Differenzen in der Wissenschaftsmethodik* bestehen. Die von Forschern wie Martini geforderte strenge Kausalanalyse behält in der psychosomatischen Krankheitsbetrachtung ihren Sinn und ihren Platz. Nicht aber umgekehrt: die Sinnfrage läßt sich nicht durch die Kausalanalyse bewältigen … Der umfassende leib-seelische Gesamtvorgang setzt *auch* Kausalität; aber das Subjekt, der höchste und aktive Integrationsort der Person ist selbst kausalanalytisch nicht adäquat faßbar. Wer sich dieser Grundeinsicht verschließt, verschließt sich einer echten Anthropologie (Mitscherlich 1949: 33).

Am Ende seines Vortrags kam Mitscherlich auf die Zielsetzung der Psychosomatischen Medizin zurück; als Ferment, „das aktiv genug ist, um wieder einmal einen Stilwandel der ärztlichen Kunst zu bewerkstelligen“, habe sie den Anspruch, „der Persönlichkeit zu einer volleren Integration, zu einer gelungenen Wesensverwirklichung zu verhelfen“ (Mitscherlich 1949: 40).

Im Anschluss an die beiden Vorträge von Weizsäcker und Mitscherlich waren nicht weniger als acht Diskussionsredner vorgesehen. Dokumente, die über Auswahl und Einladungspolitik Auskunft geben könnten, haben sich leider nicht erhalten. Einiges deutet darauf hin, dass Curt Oehme, dem als Vorsitzenden das Vorschlagsrecht für das Kongressprogramm zukam, beides ohne eingehende Diskussion im Vorstand festgelegt hatte. Im Dezember 1948, vier Monate vor dem Kongress, hatte der mit Oehme korrespondierende Martini noch keine Kenntnis von den Rednern. Gustav von Bergmann, der als erster sprach, unterstützte die beiden Vortragenden, gleichwohl auch zwischen ihm und Weizsäcker Auffassungsunterschiede bestanden. Bergmann flüchtete – wie schon in seiner Schrift *Neues Denken in der Medizin* (1947) – in Metaphern der antiken und abendländischen Ideengeschichte und wies mit Blick auf den Ausbau psychosomatischer Forschung auf die Arbeiten seines Mitarbeiters Thure von Uexküll (1908–2004) hin. Affirmativ war neben dem Zürcher Polikliniker Paul H. Rossier (1899–1976) auch Arthur Jores (1901–1982), der in der NS-Zeit verfolgt worden war und nach Kriegsende den Lehrstuhl für Innere Medizin in Hamburg übernommen hatte (Jores 1949). Jores wurde neben Uexküll der prominenteste Vertreter der internistischen Psychosomatik. Verhaltene Reaktionen bis hin zu offenem Widerspruch zeigten sich bei den Diskutanten aus Psychiatrie (Jürg Zutt, Kurt Kolle, Siegfried Haddenbrock) und Psychotherapie (Josef Meinertz).

### Martinis Replik und Mitscherlichs Schlusswort

In seiner Replik auf dem Internistenkongress bekräftigte Martini sein Bekenntnis zur potenziellen Unbegrenztheit psychophysischer Zusammenhänge in der klinischen Medizin: „Daß psychosomatische Einflüsse existieren, ist uns allen gewiß; wieweit sie reichen, ist ungewiß“ (Martini 1949a: 51). Um sich Klarheit zu verschaffen, benötige es „zuverlässige und anerkannte Methoden“. Das war das Stichwort. 1932 hatte Martini sein Manifest der Therapieforschung unter dem Titel *Methodenlehre der therapeutischen Untersuchung* niedergelegt. Darin forderte er in Anlehnung an experimentelle Vergleichsuntersuchungen und unter Zuhilfenahme statistisch-probabilistischer Methodik ein kontrolliertes Vorgehen bei klinischen Interventionen (Martini 1932; Stoll, Roelcke & Raspe 2005; Sammer & Hofer 2020). 1947 erschien die zweite Auflage mit leicht verändertem Titel: *Methodenlehre der therapeutisch-klinischen Forschung*. In dieser forderte Martini erneut wissenschaftsbasierte Methoden auf allen Gebieten klinisch-therapeutischer Forschung (Martini 1947). Vor diesem Hintergrund trat Martini der Auffassung von Weizsäcker, der methodische Sondergesetzlichkeit eingefordert hatte („die Psychosomatische Medizin kritisiert sich selbst“), vehement entgegen:

Weder eine sogenannte naturwissenschaftliche, noch eine wissenschaftliche oder eine psychosomatische Medizin können ihre eigenen Gesetze ihrer Methodologie und der Kritik ihrer Heilerfolge selbst erlassen. Diese Gesetze sind präexistent, und zwar sind es die für uns alle verbindlichen Gesetze der Logik und der Erkenntnistheorie. Diese Gesetze schließen in sich ein die Anerkennung der Kausalität. Sie ist weder eine Sache der Gewohnheit, wie Mitscherlich meint, noch eine Frage des Denkstils; sie ist nicht nur apriorisch begründet, sondern in einer unendlich großen Erfahrung. Die Medizin wird nie anders ihrer Aufgabe, kranken Menschen zu helfen, gerecht werden können, als auf der Grundlage, daß in jedem ihrer Bereiche der größtmögliche Grad von kausaler Beweisbarkeit erstrebt wird (Martini 1949a: 53).

Die Psychosomatische Medizin, so Martini weiter, habe von der Psychotherapie die Absage an strenge kausale Ansprüche übernommen – „in Deutschland in besonders radikaler Weise. Wo die Kausalität aufhört, bleibt aber immer nur Gnosis, oder das, was Weizsäcker Erlebnisgläubigkeit nennt. Dann wird auf das Streben nach Evidenz und auf den Beweis der Allgemeingültigkeit verzichtet, und es gelangt das Gefühl zu Herrschaft.“ Daran anschließend stellte er fest: „Medizinische Anthropologie soll heißen, die medizinische Lehre vom ganzen Menschen. Wenn die psychosomatische Medizin aber weiter auf die Kausalität verzichtet, wird sie nicht zur Reformbewegung, sondern nur zur Sekte werden können. Das wäre für die Medizin ein großer Verlust“ (Martini 1949a: 53f.).

Nachdem Martini und die anderen Diskussionsredner gesprochen hatten, überließ Weizsäcker das Schlusswort Mitscherlich. Dieser zeigte sich erleichtert: Die Diskussion hätte Konsens im Grundsätzlichen erkennen lassen, Kritik habe sich vor allem daran entzündet, das Subjekt in die Krankheitsauffassung miteinzubeziehen. Dies war allerdings ein gewichtiger Punkt, und Mitscherlich versuchte, ihn nochmals zu schärfen: „Wenn wir das Subjekt als agierendes, Wirklichkeit kreierendes, einmal in diese Welt unserer Wissenschaft hereingelassen haben, dann müssen wir uns darüber klar sein, daß wir es so einfach nicht wieder loswerden, um uns bei der puren Objektivität erneut zu beruhigen“ (Mitscherlich 1949: 79). In geradezu konstruktivistischer Manier führte er den Begriff der Subjektivität zudem an die Person des klinischen Wissenschaftlers heran. Auch der forschende Arzt könne die Subjektivität nicht abstreifen, sie sei in jedem Erkenntnisprozess enthalten und ein intrinsisches Moment klinisch-wissenschaftlicher Praxis: „In der Objektivierung als einem (wissenschaftlichen) Akt ist nämlich immer der enthalten, der den Akt vollzieht“ (Mitscherlich 1949: 81).

Im Nachgang des Kongresses schlug die Debatte hohe Wellen in der Fachöffentlichkeit. Sie fand zudem Fortsetzung in persönlicher Korrespondenz. Ein Beispiel ist Karl Jaspers (1883–1969), der zwar nicht Teilnehmer des Kongresses war, jedoch von Kurt Kolle (1898–1975) jenen Band der Zeitschrift *Psyche* zugesandt bekam, der die Tagungsbeiträge enthielt. Jaspers sah hinter den Vorträgen von Weizsäcker und Mitscherlich die von ihm abgelehnte Psychoanalyse durchschimmern und fand im Medium des Briefes scharfe Worte (Bormuth 2002: 233f.).^6^ Auch der Freiburger Pathologe Franz Büchner (1895–1991) bezog in seinem Vortrag *Grundsätzliches zur Psychosomatischen Medizin* Stellung. Die geforderte Verlagerung der psychosomatischen Krankheitsforschung „von der Frage nach den Ursachen auf die nach ihrem Sinn“ lehnte er ab, erkannte jedoch an, dass ein Problem der Medizin aufgeworfen sei, das nähere Betrachtung und Bearbeitung verdiene. „Als Wissenschaft wird die Medizin weiterhin an den Felsen der Naturwissenschaft geschmiedet bleiben“, resümierte Büchner – und sprach gleichzeitig von der „Aufgabe, neben den Naturwissenschaften als Grundwissenschaft der Medizin eine wissenschaftliche Anthropologie aufzubauen“ (Büchner 1951: 92).

## Kausalität und Evidenz, Statistik und Subjektivität

In einem weiteren Schritt möchte ich nun jenen Positionierungen und Differenzen nachgehen, die 1949 mit expliziten, divergierenden Auffassungen von Kausalität, Evidenz, Statistik und Subjektivität einhergingen. Diese Differenzen zeigten sich auf dem Internistenkongress in exponierter Art und Weise, sind aber gleichwohl in größeren Kontexten zu sehen. Wie im Folgenden gezeigt wird, stand die Kontroverse von Martini mit Weizsäcker in einer langen Kontinuität. Auch die Auseinandersetzung mit Mitscherlich hatte eine Vorgeschichte – und wurde nach 1949 noch über eineinhalb Jahrzehnte lang fortgesetzt. Damit wird zugleich deutlich, dass die Wiesbadener Kontroversen in epistemologische Herausforderungen und Auseinandersetzungen eingebettet waren, die es in der klinisch-wissenschaftlichen Medizin (spätestens) seit den 1920er Jahren und (zumindest) bis in die 1960er Jahre hinein gab.

Wie eingangs angedeutet, hatte Martini in der Deutschen Gesellschaft für Innere Medizin eine gewichtige Sprecherfunktion inne. Als deren erster Vorsitzender in der Nachkriegszeit hatte er 1948 deutliche Worte zur Ärzteschaft im Nationalsozialismus gefunden. Die Ursachen des „Irrwegs der Medizin“ sah Martini in einer verhängnisvollen Abkehr von abendländisch-christlichen Werthaltungen sowie moralphilosophischen Positionen der Aufklärung: „Autonom gewordenes Fortschrittsstreben […] durchbrach in der Medizin die Gesetze des ihr spezifischen Objekts, des Menschen, der immer Subjekt bleibt. Hier haben wir Ärzte in den letzten Jahren ein Damaskus erlebt, und wenig Ärzte dürften in der Welt sein, die, wenn sie sich über den Sinn der Geschichte der letzten Jahrzehnte Gedanken gemacht haben, nicht auch ihre eigenen Fundamente erzittern fühlten.“ Zugleich sprach Martini besorgte Worte in eine andere Richtung. Aus den historischen Erfahrungen und Einsichten würden „nicht immer die richtigen Folgerungen“ gezogen: „Was wir jetzt vielfach sehen, ist eine überstürzte Flucht von einem eben noch verehrten mechanistischen Weltbild, das zu einer Enttäuschung geführt hat, zu einer neuen Einseitigkeit, die bis zur Diskreditierung des kausalen Denkens führt.“ Und weiter: „Auf die bewußte Ausbildung logischer und erkenntnistheoretischer Fähigkeiten wird es ankommen müssen, […] um Unbewiesenes von Bewiesenem und Hypothetisches und Mögliches von Wahrscheinlichem und Regelhaftem zu unterscheiden“ (Martini 1948a: 3). Deutlich wird, dass Martini bereits 1948, in seiner eigenen Rede als Vorsitzender, die Frage nach den geistigen und wissenschaftlichen Grundlagen der Medizin auf die Agenda gesetzt und epistemologische Kontrastierungen vorgenommen hatte, die in Richtung der Kontroversen im Folgejahr wiesen. Eine Retrospektive muss jedoch früher einsetzen.

### Kausalzusammenhänge in Pathogenese und Therapie: Weizsäcker und Martini

Martinis Kritik an Weizsäcker geht fachöffentlich bis in die 1930er Jahre zurück und entzündete sich an dessen 1935 veröffentlichten *Studien zur Pathogenese.* „Krankheiten“, so hatte Weizsäcker programmatisch formuliert, entstünden „aus einer leidenschaftlichen Lebensbewegung. Ein Begreifen ihres Werdens hängt davon ab, ob man dieser Bewegung der Leidenschaft zu folgen vermag“. Für ein solches, dem Ursprung und Werden von Krankheiten „nachspürendes“ Unternehmen erwartete Weizsäcker nichts von einer „statistischen Ordnung“, die bloß Zufälle systematisiere, aber alles von einem Einbeziehen der Phänomene in einen „größeren Zusammenhang, […] den wir Geschichte nennen, in das also, was geschieht, und nicht in das, was Gesetz ist, was also nur möglicherweise geschieht“ (Weizsäcker 1935: 259). Anhand einer Reihe von Krankengeschichten aus seiner Heidelberger Klinik suchte Weizsäcker seine Vorgehensweise zu erläutern. Sein Hauptaugenmerk lag auf einer Gruppe von Erkrankungen, die er unter dem Begriff der Angina tonsillaris zusammengefasst hatte, und für deren Entstehen er – in Anlehnung an Anschauungsweisen der Psychoanalyse – bislang unentdeckte Bedingungen gefunden haben wollte, darunter insbesondere emotionale Erschütterungen, psychosexuelle Erregungen und seelische Kränkungen.

Im Jahr darauf reagierte Martini in der Zeitschrift *Fortschritte der Therapie* mit scharfer Kritik. Martini warf Weizsäcker vor, aus Angst vor einer am falschen Ort angewandten Statistik einen „schlimmen Fehler“ begangen zu haben, „indem er in völliger Einseitigkeit und Begrenztheit auf einen einzigen Faktor des persönlichen Geschehens – der immer und überall nur im Seelischen gesucht wird – alle Schuld (im Sinne von Ursache!) häuft“ (Martini 1936a: 255). *Dass* in der Vorgeschichte von Anginakranken emotionale und seelische „Kränkungen“ entdeckt werden könnten und ein ursächlicher, direkter Zusammenhang mit der Krankheit prinzipiell möglich sei, bestritt Martini nicht, sah für letztere Annahme aber keinerlei Belege vorgebracht. Weizsäckers Auswahl, Zusammenstellung und Interpretation nur weniger Krankengeschichten sei willkürlich erfolgt, „ohne auch nur den Versuch zu machen, zu beweisen, warum er sie nur so und nicht anders interpretieren durfte“ (Martini 1936a: 255).

Der Psychotherapeut Johannes H. Schultz (1884–1970), der Weizsäcker in vermittelnder Absicht beisprang, stellte in Zweifel, ob Martini mit seiner Kritik „das Wesentliche“ getroffen habe. Schultz räumte jedoch ein, dass für eine tiefere Erkenntnis der von Weizsäcker behaupteten Zusammenhänge „zunächst ‚botanisiert‘ gesammelt werden muß“ (Schultz 1936: 384). Daraufhin erneuerte und konkretisierte Martini seine Kritik am Beispiel der *psychogenen Angina* und insistierte auf dem „Unterschied des Möglichen und des Wahrscheinlichen in der Medizin“ (Martini 1936b: 511). Wenn Menschen sich in belastenden Lebenssituationen befänden, so Martini, vielleicht sogar in einer „Peripetie des Lebensdramas“ (Weizsäcker) und kurz darauf klinisch manifeste Symptome einer körperlichen Erkrankung zeigten, so stünden diese beiden Ereignisse in einem zeitlichen Zusammenhang; möglicherweise sogar in einem ursächlichen. Aber war ein solcher zwischen einer seelischen Erschütterung und einer – ansonsten aufgrund von Infekten auftretenden – Mandelentzündung nachweislich zu belegen? Dies war der Kern von Martinis Kritik, der jenseits des zeitlichen Zusammenhangs der Ereignisse keine Beweise für einen kausalen Nexus vorgebracht sah. Selbst wenn man diesen Zusammenhang auch nur als wahrscheinlich ansehen wolle, müssten methodische Vorbedingungen in Gestalt einer statistischen Ordnung erfüllt sein: „Ein ernsthafter Botaniker wird aus seiner Sammeltätigkeit erst dann Schlüsse auf ursächliche Zusammenhänge ziehen, wenn er so viel gesammelt hat, daß er diese Schlüsse auch beweisen kann. Nur das, nicht mehr und nicht weniger, müssen wir auch in unserem Kreis von uns und von anderen verlangen“ (Martini 1936b: 512).

Weizsäcker erwiderte die Kritik damals nicht. Angesichts der von Martini geforderten statistischen Belege zeigte er sich harthörig und lehnte sie konsequent ab. Schon 1926, auf dem ersten ärztlichen Psychotherapiekongress in Baden-Baden, hatte er hinsichtlich der Beurteilung klinischer Heilerfolge von „völlig verschiedenen Gewißheits- und Evidenzbegriffen“ gesprochen und sich eindeutig positioniert. Die „statistische Methode“, so Weizsäcker in seinem Vortrag *Psychotherapie und Klinik,* stünde im „äußersten Gegensatz zum Geiste einer Psychotherapie“ (Weizsäcker 1927: 232). Von dieser Position rückte er nicht mehr ab. In seiner Autobiografie *Natur und Geist* (1954, nach Angaben Weizsäckers großteils 1944 in Breslau verfasst) heißt es dazu:

Fortfahrend kritisierte ich also den Versuch, Heilerfolge durch Statistik festzustellen […]. Meine Abneigung gegen Statistik ist immer geblieben, und es verdroß mich, daß z. B. Martini, ein Schüler Friedrich von Müllers, gerade mit seinem Verlangen, Therapie durch Statistik zu prüfen, vielfach Gehör fand. Daran war doch nur richtig, daß man die kritiklose Anpreisung von Heilerfolgen bekämpfte; aber Statistik als die beste und womöglich einzige Form der Kritik anzupreisen, dies war doch bereits ein Anzeichen beginnender Öde im Denken und mußte als Schrittmacherei der Zahlenbarbarei wirken (Weizsäcker 1954: 155).

Weizsäckers eigene „Erkenntnistheorie klinischer Erfolgsbeurteilung und damit überhaupt klinischer Objektivität“ wollte dieser „auf der Grundlage von Personengemeinschaften“ verstanden sowie in „individuellen Konstellationen“ verhandelt wissen: „Man müsste also einen jeden Fall mit seinem besonderen Beweisgewicht anführen, und in einer Statistik sozusagen die Fälle mit einem Pluralwahlrecht ausstatten“ (Weizsäcker 1927: 233). Sein Personenbegriff sah im Wortsinn des per-sonare Wesentliches: „Das Durchtönen, Sprechen, Sichausdrücken von Menschen, das Metaphersein, das Durch-etwassein, Etwasempfinden, Sichverwandeln“ (Weizsäcker 1927: 235).

Im Vorfeld des Wiesbadener Kongresses wurden die Auffassungsunterschiede wiederholt sichtbar. Martinis Aufsatz „Kausalität und Medizin“, 1948 im ersten Band der Zeitschrift *Studium Generale* (mit dem nennenswerten Untertitel *Zeitschrift für die Einheit der Wissenschaften im Zusammenhang ihrer Begriffsbildungen und Forschungsmethoden*) erschienen, ist diesbezüglich ein Schlüsseltext. Unter Rückgriff auf die Debatte über die Pathogenese erneuerte Martini seine Kritik an Weizsäcker:

Für große Gruppen von Krankheiten ist ein wesentlicher Beitrag der seelischen Krankheitsentstehung noch nie wahrscheinlich gemacht worden. Alle Versuche, z. B. bei Infektionskrankheiten den psychogenen Faktoren eine mehr als begünstigende Rolle zuzuweisen, sind missglückt. Das heißt, sie waren unbeweisend für den, der Wert auf eine folgerichtige und schlüssige Beweisführung legt. Das aber ist die Streitfrage, die aufgeworfen ist, ob folgerichtige oder schlüssige Beweise der Medizin angemessen sind und welche Vorbedingungen sie in der Medizin haben (Martini 1948b: 346).

In seinem Aufsatz zur Kausalität gebrauchte Martini erstmals, wenn auch en passant, den Begriff der Evidenz, als Ausweis und Anspruch der „exakten Wissenschaften“ (Martini 1948b: 349). „Exakt“ meinte bei Martini die Erfüllung höchster Kausalitätsansprüche, wie sie nur den Naturwissenschaften möglich und gegeben sei. Demgegenüber sah er die Medizin in einer ungleich schwierigeren Lage, da sie „wahrhaftig nicht nur Naturwissenschaft“, sondern auch „Wissenschaft vom Menschen“ sei und ihre Subjektbezogenheit weder abstreifen könne noch dürfe (Martini 1948b: 342). Diese Sonderstellung dürfe jedoch nicht zum Verlust der Kausalität als kategorialem Forschungsprinzip führen, denn nur mit diesem könne eine Antwort auf die zentrale Frage der Klinik gefunden werden, welchen „Wert“ medizinisches Wissen habe, „was es praktisch leiste“ (Martini 1948b: 345).

Was meinte Martini mit Kausalität, was mit Evidenz? Seit dem ausgehenden 19. Jahrhundert hatte sich – vor allem im Zusammenhang mit der Herausbildung der wissenschaftlichen Bakteriologie – die Identifizierung einer notwendigen Krankheitsursache als Voraussetzung einer wirksamen Krankheitsbehandlung durchgesetzt (Gradmann & Schlich 1999). In den folgenden Jahrzehnten zeigte sich, dass mit der Etablierung von Kausalität als Ordnungsprinzip wissenschaftlich-klinischen Denkens und Handelns eine Diversifizierung einhergegangen und es zu unterschiedlichen Gesichtspunkten, Verständnissen und Gebrauchsformen gekommen war. Hierzu trugen die nach dem Ersten Weltkrieg verstärkt einsetzende Kritik an streng naturwissenschaftlichen Betrachtungsweisen sowie neue, holistisch geprägte Konzepte und Erklärungsmodelle wie die Endokrinologie oder die Konstitutionslehre bei (Metzger 2016), die ihrerseits mit neuen Leitbegriffen (wie Disposition, Funktion, Korrelation, Regulation) Krankheitsgeschehen zu ergründen suchten. Hinzu kamen Forderungen etwa nach einer stärkeren Berücksichtigung der Psychogenese von Krankheiten sowie psychotherapeutischen Ansätzen in der internistischen Klinik. Neben möglichst lückenloser Bestimmung von aufeinanderfolgenden, sich jeweils ursächlich bedingenden Mechanismen wurde auf das erklärende Verstehen von kausalen Verlaufs- und Begründungszusammenhängen verwiesen. Die unterschiedlichen Kausalitätsvorstellungen beförderten nicht zuletzt in der medizintheoretischen Reflexion eine produktive Unruhe. 1935 notierte der angehende Arzt und Medizintheoretiker Karl Eduard Rothschuh (1908–1984): „Man muß sich endlich auch in der Medizin darüber klarwerden, ob man mit der Beziehung von Ursache und Wirkung einen Sinnzusammenhang oder einen wirklichen Sachzusammenhang bezeichnet“ (Rothschuh 1935: 1404).

In seiner *Methodenlehre* hatte Martini den Begriff der Kausalität sowohl in Richtung einer mechanistischen als auch einer probabilistischen Bedeutung gerückt. Therapeutische Mittel und Methoden waren idealiter als „kausal“ zu bezeichnen, wenn „aus einer direkten Einsicht in physiologisch-pathologische Einheitszusammenhänge heraus zu handeln und willkürlich in einen solchen Zusammenhang so einzugreifen [ist], daß der normale Zustand oder doch eine so große Annäherung an ihn wieder erreicht wird“ (Martini 1947: 1). An dieses zweischrittige Vorgehen – das Erkennen des ursächlichen Zusammenhangs im Krankheitsverlauf und das Wiedererreichen des „normalen Zustands“ durch eine wirksame therapeutische Intervention – knüpfte Martini den klinischen Beweis, der mit kontrollierten Versuchsanordnungen und unter Einsatz statistischer und probabilistischer Methoden zu erbringen war. Kausalität und klinischer Beweis bedingten einander; das eine war ohne das andere nicht denkbar. Deutlich wird nun auch, warum Martinis Verständnis von Kausalität zu einem Evidenzbegriff in Richtung der Bedeutung einer rigorosen Beweisführung, eines exakten Beweises drängte.

Martinis Evidenzverständnis war hierbei auch historisch grundiert. Carl Wunderlich (1815–1877) hatte bereits 1851 in seiner klinischen Antrittsvorlesung in Leipzig gefordert, die Wirksamkeit von therapeutischen Interventionen „evident zu machen“ (Wunderlich 1851: 109). Wunderlichs rationale Therapie kritisierte unkontrollierte ärztliche Erfahrungen als „Reminiszenzen des Selbsterlebten“ und forderte das Erbringen von „Beweisen“ unter Zuhilfenahme statistischer Mittel. 1949 rief Martini Wunderlichs modern anmutenden Evidenzbegriff in Erinnerung und setzte ihn erneut auf die klinisch-wissenschaftliche Agenda. Evident war nicht das Erlebte und Erinnerte, sondern das Bewiesene; das nach methodisch valider Prüfung zur Gewissheit Gebrachte (Martini 1949a: 53; Martini 1949b).

Gleichwohl gebrauchte Martini den Evidenzbegriff nicht nur im Sinne von empirisch nachprüfbaren, intersubjektiven, statistisch grundierten Belegen, sondern auch mit anderen Bedeutungskomponenten. Gegenüber der von Weizsäcker und Mitscherlich betonten ärztlichen Subjektivität, die jedem Handeln, auch ärztlichem Forschungshandeln, innewohne, blieb er distanziert, ließ jedoch Nachdenklichkeit erkennen. Dies wird in seinem Einführungsvortrag *Grundsätzliches und Methodisches zur therapeutisch-klinischen Forschung *auf dem ersten Therapiekongress in Karlsruhe (1949) deutlich:

Die Evidenz, die der Arzt als Forscher erreichen kann, schließt fast immer solche subjektiven Momente in sich, weil nichts Ärztliches sein kann, was nichts Subjektives an sich hat. Ebenso sicher ist auch, daß diese Evidenz vom Subjektiven aus immer in ihrer Zuverlässigkeit bedroht ist […]. Die in der Mitwirkung des Seelischen begründete Subjektivität ist die immer drohende Klippe unserer Beweisführung. Sie ist der Urgrund der schwierigsten Problematik unserer Arbeit, sie ist das Problem schlechthin: denn sie ist die Ursache der beglückenden Doppelgesichtigkeit der Medizin, die auf Körper und auf Geist-Seele gerichtet ist (Martini 1949b: 1352).

Auch Weizsäcker gebrauchte im Vorfeld der Wiesbadener Begegnung den Begriff der Evidenz: „Es scheint mir nicht richtig zu sein, die psychologische Methode als unexakt der Naturwissenschaften gegenüberzustellen“, hieß es in seinem Aufsatz *Über das Wesen des Arzttums*. Und weiter: „Sie hat nur einen anderen Evidenzcharakter, und wir müssen zugeben, daß die Psychologie heute einen viel unmittelbareren Zugang zur Bestimmung des Menschen bietet als die Naturwissenschaft“ (Weizsäcker 1947/1950: 196). In seinem Wiesbadener Vortrag griff Weizsäcker den Evidenzbegriff nicht explizit auf, wiederholte jedoch sein Plädoyer für eine am Subjekt orientierte Medizin, die ihren methodischen Standort ändern und um die Erkenntnismöglichkeiten der Tiefenpsychologie erweitern müsse: „Als wahr soll nur gelten, was man entscheiden kann, indem man es verändert“ (Weizsäcker 1949b: 14). Diesen Veränderungsimpetus richtet Weizsäcker vor allem an die internistische Klinik. In dieser müsse Seelisch-Unbewusstes „mit gleicher Akkuratesse und Kritik erforscht werden wie der Körpervorgang“. „Laboratoriumsapparatur“ und „statistische Erhebungen“ kämen dafür nicht infrage; die Klinik bedürfe der Psychologie. Erst mit ihrer Hilfe könne Seelisches und Körperliches in seinen „verborgenen Umgangsformen“ aufgesucht werden: „Die Anstrengung dies Tiefliegende zutage zu bringen heißt Wissenschaft“ (Weizsäcker 1949b: 16).

### Freiheit der Betrachtungsweisen? Mitscherlich und Martini

Für den 1908 – und damit im gleichen Jahr wie Thure von Uexküll – geborenen Alexander Mitscherlich war es ein großer Erfolg, auf dem bedeutendsten Medizinerkongress des deutschsprachigen Raumes sprechen und die Anliegen der Psychosomatischen Medizin vorbringen zu können. Klinisch-praktisch bei Curt Oehme, theoretisch-wissenschaftlich bei Viktor von Weizsäcker geschult, war Mitscherlich vor allem durch seine Rolle beim Nürnberger Ärzteprozess bekannt geworden. Das Fundament seines Vortrags bildete jedoch Mitscherlichs 1946 veröffentlichte Schrift *Freiheit und Unfreiheit in der Krankheit*.

Mitscherlich hatte diese Schrift im letzten Kriegsjahr in Heidelberg verfasst. Sie war seinen Angaben zufolge aus Notizen entstanden, die er zwischen überfüllten Sprechstunden in der Klinik und abendlichen Selbstreflexionen am Schreibtisch angefertigt hatte – „tastende Versuche“ auf dem Weg zu Antworten auf die alte, erneut dringlich gewordene Frage „Was ist der Mensch?“. Als Paten dieser Schrift nannte Mitscherlich neben Sigmund Freud, „dessen Tiefblick auch die Finsternis der vergangenen Jahre zu durchwandern half“, Curt Oehme, der ihm die „unbestechliche Exaktheit der Naturwissenschaften anschaulich gemacht und durch gütige Kritik immer geholfen“ habe (Mitscherlich 1946/1983: 16).

In der unmittelbaren Nachkriegszeit – das zeigen Briefe aus dem Nachlass von Mitscherlich – standen die beiden im engen Kontakt, der an eine symbiotische Beziehung heranreichte. Mitscherlich war für Oehme ein wichtiger Gesprächspartner, der nicht nur Vorträge und Aufsatzentwürfe kommentierte, sondern auch über seine Kontakte zur US-amerikanischen Militärregierung Rat erteilen sowie kraft seines Zugangs zu „Schweizer Ressourcen“ auch materielle Unterstützung gewähren konnte (Na 7, 121: Oehme an AM, 16.05. und 29.05.1946; AM an Oehme, 03.05.1947). Oehme wiederum agierte bei Mitscherlichs Bemühungen in der unmittelbaren Nachkriegszeit um die Etablierung einer Abteilung für Psychotherapie beziehungsweise Psychosomatik in der Heidelberger Fakultät als dessen Fürsprecher und war 1947 in den Auseinandersetzungen, die Mitscherlich im Zuge der Dokumentation des Nürnberger Ärzteprozesses mit Franz Büchner, Wolfgang Heubner (1877–1957) und Ferdinand Sauerbruch (1875–1951) hatte, vermittelnd eingesprungen.^7^ Vor diesem Hintergrund überrascht nicht, dass Oehme um die Jahreswende 1948/49 Mitscherlich zu einem Hauptreferat auf dem Wiesbadener Kongress einlud; angesichts der „überall spürbaren Ablehnung meiner Person und der Sache wirklich eine tapfere Tat des alten Oehme“, wie Mitscherlich es in einem Brief an den mit ihm befreundeten Schweizer Mikrobiologen Hubert Bloch (1913–1997) ausdrückte (Hoyer 2008: 185).

Mit den Leitbegriffen „Krankheit, Freiheit, Einzelner“ zeichnete Mitscherlich Umrisse einer anthropologischen Psychotherapie, die sich von der „Anciennität der Lehren“ emanzipieren und zu einer eigenständigen wissenschaftlichen Identität finden sollte. Eine Einordnung dieser Schrift fiel ihm später selbst schwer. Teile davon hatten einen aphoristischen, skizzenhaften Charakter, andere Fragmente einer Medizintheorie. Vorsichtig formulierte, aus klinisch-ärztlichen Beobachtungen gewonnene Einsichten wechselten mit selbstbewusst formulierten, politisch-epistemologischen Parolen. Für das Verständnis von Mitscherlichs Vortrag von 1949 und der mit Martini ausgetragenen Differenzen ist diese Schrift gleichwohl von großer Bedeutung, da sie Mitscherlichs argumentatives Fundament bildete. Martini wiederum, der diese Schrift früh wahrgenommen hatte, war über den Inhalt erheblich irritiert – und hatte Mitscherlich schon im Vorfeld der Wiesbadener Kontroverse als Adressaten seiner Sorge und Kritik ausgemacht. Mitscherlich seinerseits hatte Martinis Kongressrede von 1948 rezipiert.^8^

Dabei gingen Mitscherlich und Martini durchaus von ähnlichen Voraussetzungen aus. Die Sonderstellung des Menschen, seine Unvergleichbarkeit war für beide nicht verhandelbar: „Zum Menschen gibt es keinen Übergang“ (Mitscherlich 1946/1977/1983: 18). Ebendies hatte auch Martini in seiner Vorsitzendenrede festgehalten und zu einer der Lehren erklärt, die aus der NS-Vergangenheit zu ziehen waren. Differenz wird dort erzeugt und manifest, wo die übergangslose, solitäre Stellung des Menschen auf eine Medizin trifft, die „Wissenschaft vom Menschen“ sein will. Mitscherlich sah nicht nur die Psychotherapie in zu großer Nähe zur Inneren Medizin; die Heilkunde selbst habe ihre Auffassung vom Menschen mit jener der naturwissenschaftlichen Forschung zu sehr vermengt und sich deren „grundsätzlicher Exklusivität und Intoleranz“ ausgeliefert. Davon ausgehend formulierte Mitscherlich weitreichende methodisch-programmatische Konsequenzen: In der „neuen Epoche der Selbstwandlung“ müsse der Mensch seine Freiheit in allen Weisen seiner Existenz, auch in der Krankheit, zurückgewinnen. Dies bedeute, das Subjekt als Apriori *jeder *Wissenschaft vom Menschen anzusehen: Es könne nicht in einem „additiven Verfahren“ hinzugefügt, sondern müsse in der methodischen Anschauung miterfasst werden: „Wo man das Subjekt sieht, muss man auch seine Geschichte sehen“ (Mitscherlich 1946/1977/1983: 45). Damit war Mitscherlich nahe bei Weizsäcker, ebenso mit seiner Forderung nach Anerkennung einer epistemologischen Autarkie der Psychosomatischen Medizin im Rahmen eines „Pluralismus der Methoden“ (Mitscherlich 1946/1977/1983: 50). Mit dieser Argumentation immunisierte Mitscherlich seinen Standpunkt – und konnte jederzeit zum (Gegen‑)Angriff übergehen: Wer mit dieser Sichtweise nicht mitging, habe sich aus der „geschlossenen Weltsicht“ der Naturwissenschaften nicht befreien können und war „im Zeitalter des Kausalismus“ zurückgeblieben (Mitscherlich 1946/1977/1983: 60).

Die Differenz zu Martini bestand nicht nur auf inhaltlicher, sondern auch auf biografischer und politisch-institutioneller Ebene. 1949 blickte Mitscherlich auf herausfordernde Jahre zurück – und hatte sich mehrere berufliche Rollen und Identitäten zugelegt: Arzt, Publizist, Kontaktperson der US-amerikanischen Militärregierung, Beobachter des Nürnberger Ärzteprozesses und federführender Herausgeber der Prozessdokumentation sowie intellektueller Wortführer der politischen Linken (Dehli 2007: 124–144).^9^ Demgegenüber war Martini ein katholischer Universitätsmediziner, etablierter Klinikdirektor sowie Reorganisator und Sprecher der internistischen Fachgesellschaft nach dem Krieg. Ausgehend von der therapeutischen Forschung wollte Martini die klinische Medizin als Wissenschaft begreifen, ihr methodische Fundamente und Konturen geben. Von 1949 an war er am politischen Puls der Bundesrepublik. Als Begleitarzt und Berater von Bundeskanzler Konrad Adenauer war er an der Neugründung von Forschungs- und Wissenschaftsorganisationen beteiligt, darunter an der Fusion der (alten) Notgemeinschaft mit dem Forschungsrat zur Deutschen Forschungsgemeinschaft, die 1951 zustande kam (Orth 2011: 46).

1957 wurde Martini Gründungsmitglied des Wissenschaftsrats und rief die „Klinische Kommission“ ins Leben. Manche Pläne zur Stärkung der Klinischen Forschung verließen das Planungsstadium nicht. Anderes gelang, darunter die Durchsetzung der Entscheidung, an Medizinischen Fakultäten Institute für medizinische Statistik einzurichten (Hofer 2019: 49). Weitere Institute zwischen Freiburg (1963) und Kiel (1964) folgten. 1975 übernahm Martinis ehemaliger Doktorand, Hans-Joachim Jesdinsky (1931–1986), den Lehrstuhl in Düsseldorf. Das von Jesdinsky federführend erarbeitete *Memorandum zur Planung und Durchführung kontrollierter klinischer Therapiestudien* gab 1978 in der Bundesrepublik den entscheidenden Anstoß zur strukturierten Förderung von klinischer Forschung und Therapiestudien (Schumacher 2016).

### Forschungsprojekte und fortdauernde Kontroversen

Die Wiesbadener Begegnung war ein erster Höhepunkt in der Kontroverse zwischen Martini und Mitscherlich; sie nahm in der Folge weiter an Fahrt auf und setzte sich auf allen Ebenen fachöffentlicher Kommunikation fort. Die im Modus von Kritik und Entgegnung verfassten Aufsätze enthielten beiderseits Polemiken, zogen aber keinen Gesprächsabbruch nach sich. Eine gewisse Selbstimmunisierung, auch ein Orthodoxwerden der jeweiligen Positionen, lässt sich dabei nicht von der Hand weisen. 1951 etwa wiederholte Mitscherlich die Forderung nach einer anthropologischen Selbstverortung der Psychosomatischen Medizin, die zugleich Wegbereiter einer neuen ärztlichen Wissenschaft vom Menschen sein müsse (Mitscherlich 1951). Martini hingegen sah Mitscherlich vom „dionysischen Eifer der Ganzheitsmedizin befallen“ und kritisierte, dass die Psychosomatische Medizin eine zu einseitige Hinwendung zu Subjekt, Sinn und Symbol vollzog – und die Unterscheidung des Möglichen vom Wahrscheinlichen aufgegeben habe: „Die Beweisführung, die uns die Psychotherapeuten bisher zugemutet haben, sind in Wirklichkeit nicht neue Denkmethoden, sondern unzulängliche Methoden“ (Martini 1951: 162). Damit waren die Demarkationen erneut gesetzt.

### Hypertonie und Hypnose: Das Bonner „Psychotherapie-Projekt“

Um die methodischen Fragen zur Psychosomatischen Medizin in seinem eigenen Wirkungskreis bearbeiten zu können, nahm Martini Kontakt mit dem Heidelberger Physiologen Hans Schaefer (1906–2000) und dessen Mitarbeiter August Wilhelm von Eiff (1921–1998) auf. Eiff, der nach seiner medizinischen Promotion an Schaefers Institut eine vertiefte Ausbildung in experimenteller Physiologie erhalten hatte, verfügte zudem über Erfahrungen mit der Ausübung hypnotischer Techniken. Seit 1949 führte Eiff unter Anleitung von Schaefer experimentelle Untersuchungen zum Einfluss der Hypnose auf Temperaturempfindung und Wärmeregulation durch (Eiff 1951). Schaefer erhoffte sich von diesen Untersuchungen, den „Einfluss hypnotischer Effekte auf regulative Einrichtungen des Körpers exakt festzustellen und damit das große Kapitel der Beeinflussung organischer Vorgänge durch unangreifbare Untersuchungen zu bearbeiten“, wie er 1952 in einem gemeinsam mit Martini verfassten Schreiben an die DFG festhielt (DFG-Archiv, Ei 20/1). Bald wechselte Eiff zu Martini an die Bonner Klinik, um dort weitere Fragestellungen zu bearbeiten.

1952 begannen Martini und Eiff mit Förderung der DFG den „Einfluss der Psyche auf die Pathogenese der Stoffwechselerkrankungen“ mit vor allem kalorimetrischen Experimenten zu erforschen (DFG-Archiv, Ei 20/1–3). Zwei Jahre später wurde eine Verlängerung des Forschungsprojekts beantragt. Im Fachausschuss der DFG fanden die Anträge jeweils breite Zustimmung, da mit Martinis Klinik und seinen methodischen Kompetenzen „Gewähr der Exaktheit der Untersuchungen in ganz besonderem Maße gegeben“ sei und, in den Worten des Fachausschussvorsitzenden, „man auf dem mit dem Schlagwort bekannten Gebiet der Psychosomatik endlich aus dem Niveau des ‚fröhlichen Plauderns‘ herauskommen müsse“ (DFG-Archiv, Ei 20/4, 03.01.1955). 1957 schließlich schloss Eiff das Projekt mit der Veröffentlichung der Monografie *Grundumsatz und Psyche *ab, die zugleich seine Habilitationsschrift an der Medizinischen Fakultät der Universität Bonn war (Eiff 1957). Während Eiff die darin präsentierten Untersuchungen zum Energieumsatz als Nachweis von psychischen Einflüssen auf Stoffwechselveränderungen wertete, ebenso die auf dem Wege hypnotischer Appetitsuggestionen erzielte Gewichtszunahme bei einer magersüchtigen Patientin, kam aus Mitscherlichs Heidelberger Abteilung Kritik. Helmut Thomä (1921–2013), der Oberarzt von Mitscherlich, bemängelte die Vernachlässigung psychogener Ursachenforschung und die „primitive“ Suggestionshypnose, mit der man im geschilderten Fall der Patientin mit Anorexia nervosa „kaum zu einer differenten und kausal wirksamen Psychotherapie kommen“ könne (Thomä 1958: 753).^10^ Mitscherlich wiederum warf Eiff „Wunschdenken, in Theorie gekleidet“ und „Naivität in der selbstgeschaffenen Situation […] eines organmedizinischen Handlungsmodells“ vor (Mitscherlich 1958: 731).

Zu diesem Zeitpunkt hatten Eiff und Martini mit weiteren psychophysiologischen und klinisch-therapeutischen Forschungsvorhaben begonnen. Im Fokus stand nun die essenzielle Hypertonie, deren Beeinflussung durch psychodynamische Faktoren sowie die Frage der Wirksamkeit von unterschiedlichen psychoanalytischen Behandlungsverfahren. Hierzu erhielt Martini 1957 wiederum Fördermittel seitens der DFG wie auch Bundesmittel aus dem Forschungsfonds des Bundeskanzleramts (DFG-Archiv, Ma 24/12; BAK, B 136/921). An dem Vorhaben arbeiteten an der Bonner Klinik mehrere Psychotherapeuten mit, darunter Gerhard Kloska sowie Hans und Magda Quint. Die Genannten zählten 1958 zu den Gründungsmitgliedern des Instituts für analytische Psychotherapie im Rheinland. In beratender Funktion konnte Werner Schwidder (1917–1970) aus Tiefenbrunn/Göttingen gewonnen werden (Eiff 1957: 59). „Es ist eigentlich nur ein Ziel“, schrieb Martini 1957 an den Hamburger Psychoanalytiker Ulrich Ehebald (1921–2010), nämlich „der Psychotherapie eine Möglichkeit der konkreten Bewährung an einem Problem zu geben. Dabei sollen einerseits die Arbeitsmöglichkeiten der Psychotherapie voll gewahrt, andererseits die Voraussetzungen eines meinetwegen statistischen klinischen Beweises innegehalten sein“ (Martini an Ehebald, 09.07.1957, NL Martini).

Die über mehrere Jahre angelegten Untersuchungen an insgesamt 19 Patientinnen und Patienten, die „in ihrem individuellen Krankheitsverlauf“ untersucht und in definierten Zeitabschnitten psychotherapeutisch behandelt wurden, stießen allerdings auf eine Vielzahl von Schwierigkeiten. Bei mehreren Patienten verschlechterte sich der Krankheitsverlauf. Dies machte zusätzliche medikamentöse Behandlungen außerhalb des Untersuchungsdesigns notwendig. Zwei der Patienten verstarben. Hinzu kamen organisatorische Unsicherheiten und Verzögerungen, die einerseits mit der Emeritierung von Martini (1957) zu tun hatten, andererseits mit Finanzierungsschwierigkeiten, da nach zwei Jahren der bei der DFG gestellte Verlängerungsantrag nicht vollumfänglich bewilligt wurde. Schließlich zeigten sich innerhalb des Forschungsteams Unstimmigkeiten in der Frage, welche testpsychologischen Verfahren zur Beurteilung der Ergebnisse der Psychotherapie heranzuziehen waren (Martini an Hess/DFG, 13.03.1959, BAK B136/921; Martini 1959: 1291; Eiff 1967).^11^

### Psychotherapie: kollektive Forschungsmethode und der „individuelle Fall“

Vor diesem Hintergrund flammte Ende der 1950er Jahre die Methodenkontroverse neu auf. Während Martini 1958 auf dem Karlsruher Therapiekongress die Konturen einer rationalen Therapie zeichnete und vergleichend-statistische Untersuchungen auch für die Psychotherapie forderte (Martini 1958), wies Mitscherlich dies zurück und warf ihm „überzogenes Ordnungsstreben“ vor. Martinis Theorie der rationalen Therapie stünde zwar „in Kongruenz mit tatsächlichen Wirkungszusammenhängen des Naturgeschehens“, habe als klinisch-wissenschaftliches Denk- und Handlungsmodell jedoch nur begrenzte Reichweite, da es allein auf die körperliche Organisation des Menschen bezogen sei. Zudem könne Martinis „kollektive Forschungsmethode“ nur für Gruppen von Krankheiten Geltung beanspruchen, nicht aber für Individuen, deren subjektives Erlebnisgeschehen und soziale Realitäten ausgeklammert blieben. Demgegenüber forderte Mitscherlich, die Arzt-Patienten-Begegnung zum Ausgangspunkt weiterer Forschung zu machen. Die klinische Medizin müsse „selbstkritisch Methoden entwickeln und lehren, die den Arzt anweisen, wie er mit dem Kranken umgehen muß, um von ihm inhaltlich die Evidenz zu bekommen, daß er ein Mitmensch ist“ (Mitscherlich 1958: 729).

Martini wies ein Jahr später die Kritik von Mitscherlich zurück. Er konzedierte, dass zwar die „unwissentliche Versuchsanordnung“ in der Psychotherapie nicht durchführbar sei, wohl aber der Vergleich von psychotherapeutischen mit nicht-psychotherapeutischen (medikamentösen) Behandlungen bei zumindest zwei Patienten mit der gleichen Krankheit. Am Postulat, in der (psycho-)therapeutischen Forschung auf „die Häufung regelhafter Erlebnisse bei ähnlichen Alterationen und Situationen“ zu achten und diese statistisch zu erfassen, hielt Martini fest, zumal Mitscherlich selbst festgestellt habe, „daß es psychische Vorgänge gibt, die so regelhaft verlaufen, daß […] treffend von psychischen Mechanismen gesprochen“ werden könne (Martini 1959: 1291; Mitscherlich 1958: 723).

Als 1959 der Verlängerungsantrag zum Bonner Hypertonieprojekt nach informeller Mitteilung über „scharfe Kritik von Mitscherlich“ seitens des DFG-Referenten für Medizin, Günter Latsch, zu scheitern drohte, erhob Martini energischen Protest. Gegen „die sich abzeichnende Tendenz“, internistisch-psychosomatische Forschungsanträge „unter die Kritik der Psychoanalytiker“ zu stellen, „würde nicht nur ich mich mit der letzten Energie, sondern dagegen würde sich die ganze rational denkende Medizin zur Wehr setzen“ (Martini an Latsch, 27.04.1959, NL Martini). Thure von Uexküll gegenüber beklagte Martini die „krasse medizinische Unwissenheit“ von Mitscherlich, die in der Beurteilung psychotherapeutischer Methoden an Patienten mit körperlichen Erkrankungen zu „gefährlichem Optimismus“ geführt habe. Uexküll war auf Vermittlung bedacht, ließ aber vorsichtige Distanz zu Mitscherlich erkennen (Martini an Uexküll, 26.05.1959, NL Martini; Freimüller 2007: 221).^12^

Martini betonte seinerseits in brieflicher Korrespondenz sein Interesse an weiterer Zusammenarbeit mit ärztlichen Psychotherapeuten.^13^ Er beobachtete die weitere Entwicklung genau, bat um Zusendung von Tagungsberichten und Sonderdrucken und korrespondierte mit der (1949 gegründeten) Deutschen Gesellschaft für Psychotherapie und Tiefenpsychologie (DGPT) und deren Schriftführer, Tobias Brocher (1917–1998).^14^

1959, zehn Jahre nach Wiesbaden, veröffentlichte Martini seinen Artikel „Zur Frage der therapeutischen psychosomatischen Forschung“. Darin wiederholte er seine Forderung nach einem kontrollierten Vorgehen in der psychotherapeutischen Forschung und danach, insbesondere den einmal eingeschlagenen Behandlungspfad mit (vorab definierten) Beobachtungsperioden nicht zu verlassen, suggestive Mitbeeinflussungen zu separieren und psychische Vorgänge, die eine Regelhaftigkeit („psychische Mechanismen“) erkennen ließen, zu dokumentieren (Martini 1959: 1290). Martinis neuerlicher Vorstoß im Vorfeld des dritten DGPT-Kongresses, der 1960 in Verbindung mit dem Internistenkongress abgehalten werden sollte, war taktisch klug platziert. Aufseiten von Brocher und Mitscherlich, der zu diesem Zeitpunkt den Vorsitz der DGPT innehatte, führte dies dazu, den Austausch mit Martini zu suchen und eine Einladung auszusprechen. Martinis Teilnahme war an einem Round-Table-Gespräch vorgesehen, das der „Theorie der Psychosomatik und der Psychotherapie des praktischen Arztes“ gewidmet war (mit den weiteren Teilnehmern Michael Balint, Annemarie Dührssen, Arthur Jores, Alexander Mitscherlich, Werner Schwidder und Thure von Uexküll). Martini nahm die Einladung mit dem augenzwinkernden Hinweis an, dass Weizsäcker und Mitscherlich 1949 auf dem Internistenkongress „große Referate zugebilligt“ worden seien und „diesmal wenigstens ein Advocatus diaboli zu Wort kommen“ müsste (Martini an Brocher, 07.04.1960; NL Martini).

Das Leitthema des DGPT-Kongresses war in Anlehnung an Michael Balints (1896–1970) Buch *Der Arzt, sein Patient und die Krankheit* (1957) gewählt worden: *Zur Psychotherapie durch den praktischen Arzt*. Martini hatte Balints Buch genau gelesen und annotiert. Er signalisierte in seinem Redebeitrag partielle Zustimmung, warf dem anwesenden Balint jedoch eine Überdehnung des Neurosebegriffs, die Missachtung der körperlichen Differenzialdiagnose, den Verzicht auf Kontrollinstanzen in der Therapie und nicht zuletzt das Jonglieren mit suggestiven Begriffen vor. Wenn sich praktische Ärzte mit Psychotherapie beschäftigten, bestünde die Gefahr, „dass auch die Praktiker in die Versuchung kommen, ihre ‚trügerischen Erfahrungen‘ zu publizieren, und es könnte so erst recht eine Scheinwissenschaft zu Papier gebracht werden“ (Martini, Redemanuskript 1960, NL Martini).

Gegen den daraufhin erhobenen Vorwurf, die Psychotherapie grundsätzlich abzulehnen, verwahrte sich Martini jedoch: „Mein Anliegen ist nicht die Psychotherapie zu bekämpfen. Manche von Ihnen werden sich schwertun, mir das zu glauben. Aber habe ich denn die sogenannte Schulmedizin bekämpfen wollen, seit ich seit 30 Jahren immer wieder auf die Fehler ihrer Beweisführung, besonders in der Therapie, hinwies?“ (Ebd.) Dieses Argument war in der Sache kaum zu entkräften, da Martinis Methodenkritik nachweislich sowohl programmatisch als auch pragmatisch die Behandlung körperlicher Krankheitsbilder in der medizinischen Klinik adressierte. In seinem eigenen fachlichen Umfeld, der Inneren Medizin, wurden Martinis fortgesetzte Weckrufe zur rigorosen Prüfung therapeutischer Interventionen zwar gehört, jedoch nur verhalten in die klinische Forschungspraxis integriert. Die von Arthur Jores konstatierte „Erfolglosigkeit“ seiner Methodologie wusste Martini dennoch zu kontern. Dieser habe „großenteils recht, wenn er nur auf Deutschland sieht. Aber in der ausländischen Literatur sieht es anders aus; wenn man sie genau betrachtet, dann erkennt man, daß wir auch auf diesem Gebiet – dem der Kritik der therapeutischen Beweisführung – zurückgeblieben sind.“^15^ Mit dem Hinweis auf die vorausgeeilte internationale Forschung suchte Martini die Legitimität seiner eigenen Position zu stärken sowie bereits erhobenen Forderungen neues Gewicht zu verleihen. Die größte Gefahr sah er nach wie vor in einem therapeutischen Vorgehen, das von Fall zu Fall variiere und ohne vorab definierte Kontrollinstanzen blieb. 1961 reagierte er auf die Zusendung eines Separatums von Mitscherlich über die Behandlung eines Patienten mit einem chronischen Ekzem (Mitscherlich 1961) mit der Schilderung eines ganz ähnlichen Falls, um pointiert festzuhalten: „Sie haben hier **ein** Erlebnis, ich habe hier gleiches Erlebnis. Der Unterschied zwischen uns ist aber, daß Sie aus einem Erlebnis schon Schlüsse ziehen, und daß ich das für irreführend halte“ (Martini an Mitscherlich, 14.09.1961).

Seit der ersten Auflage seiner *Methodenlehre* 1932 hatte Martini gefordert, der Kasuistik ein gesichertes Fundament zu geben. Das Verhältnis von „Kasuistik und Statistik in der Medizin“ stellte er in den Mittelpunkt einer seiner letzten Veröffentlichungen (Martini 1961). Darin zeigte er sich problembewusst gegenüber den Grenzen der Statistik, die nur dort zum Einsatz kommen könne, wo die Bildung von zwei homogenen Vergleichsgruppen von Kranken möglich sei: „Der einzelne Kranke, die einzelnen Glieder der beiden Gruppen gehen bei solchem statistischen Vorgehen niemals als selbstständige Individuen in die Rechnung ein, sondern nur als Glieder von Kollektiven“ (Martini 1961: 2). Davon ausgehend rief er die Bedeutung der Kasuistik für den kontrollierten „individuellen therapeutischen Vergleich“ in Erinnerung. Wolle man aus einem „Casus“ folgerichtige und nachweisliche Schlüsse ziehen, so müsse der Vergleich in die individuelle Krankengeschichte hineingetragen werden. Hierbei käme es darauf an, dass der einzelne Fall so geordnet wird, dass er hinterher in seinen zeitlichen Zusammenhängen überschaubar ist, denn so wie sich

bei dem kollektiven Vergleich die beiden Gruppen von Kranken nur in einer Hinsicht, nämlich in Bezug auf das zu prüfende Medikament, unterscheiden durften, so dürfen sich jetzt im individuellen Vergleich auch die neuen Vergleichspartner, das sind die zu vergleichenden zeitlichen Perioden – schematisch geordnet in Vorbeobachtungszeit, Zeit der therapeutischen Prüfung und eventuell Nachbeobachtungszeit – durch nichts anderes unterscheiden als einzig und allein durch das eine zu prüfende Heilmittel (Martini 1961: 3).

Auf diese Art und Weise ließe sich auch bei einem individuellen Fall nachprüfen, ob „ein ursächlicher Zusammenhang zwischen dem Krankheitsverlauf und den ihn möglicherweise bestimmenden Faktoren wahrscheinlich gemacht werden kann“ (Ebd.).

Eingehend suchte Martini das Verhältnis von Kasuistik und Statistik zu ergründen. Sie waren für ihn keine Konkurrenten, sondern für die Gewinnung therapeutischer Erkenntnisse gleichermaßen unentbehrlich. Bemerkenswert war schließlich seine Feststellung, dass der individuelle therapeutische Vergleich „grundsätzlich dem Menschen adäquater sei als dessen Einordnung in ein Kollektiv“. Mit dieser Vorgehensweise war er nahe an den heutigen *n‑of-1-trials*, die in Ergänzung zu gruppenbezogenen klinischen Studien und Metaanalysen ein kontrolliertes therapeutisches Vorgehen am einzelnen Patienten ermöglichen. Das seine eigenen Forschungen zusammenfassende Kapitel „Die therapeutische Prüfung im Bereich der psychosomatischen Erkrankungen“, vorgesehen für die vierte Auflage seiner *Methodenlehre*, konnte Martini, der 1964 verstarb, nicht mehr realisieren. Es blieb Fragment und fand in dem vier Jahre später von seinen Mitarbeitern herausgegebenen Werk keine ausdrückliche Berücksichtigung (Martini et al. 1968).

## Schlussbetrachtungen

Dass das Jahr 1949 Referenzpunkt für die weitere methodologische Diskussion blieb, zeigt ein Ausblick auf den gemeinsam von DGIM und DGPT ausgerichteten Kongress von 1967. Die Vorsitzenden Arthur Jores (DGIM) und Werner Schwidder (DGPT) brachten das Thema *Methodologie in der Psychosomatik* erneut auf die Agenda, um kooperativen Ansätzen Raum und Repräsentation zu geben. Jores wollte damit „zeigen, daß der gewiß im Jahre 1949 noch etwas schwankende Boden der Psychosomatik inzwischen wesentlicher fester geworden ist“ (Jores 1967: 4). Das Spektrum der Hauptvorträge umfasste klinisch-wissenschaftliche Untersuchungsmethodik (Joannes Juda Groen), Psychoanalyse (Adolf-Ernst Meyer), psychophysiologische Testverfahren (Lennart Levi, August Wilhelm von Eiff), soziale Epidemiologie (Manfred Pflanz) sowie Berufswelt und Krankheit (Paul Christian). Vieles war anders geworden – und doch zeigte sich eine diskursive Pfadabhängigkeit, die auch in der Folge blieb. Bis in die 1990er Jahre firmierte der 1949er-Kongress als Fixpunkt im internistischen Fachgedächtnis, als exemplarischer Ort der Auseinandersetzung um die Wissenschaftlichkeit der klinischen Medizin (siehe z. B. Köbberling 1997).

Allerdings stand schon 1949 die Debatte über die Psychosomatische Medizin im Sog von epistemologischen Fragen, die bereits in den 1920er Jahren offen zutage getreten und über die politischen Brüche hinweg weitgehend ungelöst geblieben waren. Diese Einschätzung wurde von allen Akteuren der Kontroverse selbst zum Ausdruck gebracht, zum Teil unter Berufung auf gleiche begriffliche Topoi (wie „Krise der Medizin“; Mitscherlich 1949: 24; Roelcke 2016). Hinter den Pointen der Vortragenden und mancher Polemik verbarg sich inhaltlich kaum Neues oder Überraschendes. Der Kongress von 1949 hatte zweifelsohne historisches Momentum; gleichzeitig lässt er sich als Kulmination in einer Kontinuität von methodisch-epistemologischen Kontroversen lesen, die über Jahrzehnte andauerten.

Der Kongress und die fortgesetzten Kontroversen hinterließen bei allen Beteiligten Spuren, auch bei Martini. Im Vergleich zu seinen früheren, experimentell und pharmakotherapeutisch geprägten Arbeiten öffnete er sich nach 1949 anthropologischen Perspektivierungen auf die Beziehung von „Arzt und Kranker“ (Martini 1953). Eine größere Nachdenklichkeit zeigte er auch in der Frage, mit welchen Schwierigkeiten die klinisch-therapeutische Forschung aufgrund der „übergroßen Vielfältigkeit des Menschen in seiner Krankheit“ konfrontiert war, konfrontiert sein musste (Martini 1962: 753; Martini 1963). Deutlich ist weiterhin, dass Martini sich in der Forschung nachhaltig der Psychosomatischen Medizin und ärztlichen Psychotherapie (im Haus und Gefüge der internistischen Klinik) zuwandte. Dies belegen nicht nur die von ihm angestoßenen und an seiner Bonner Wirkungsstätte verfolgten Projekte zur Psychophysiologie und Psychotherapie der Hypertonie, sondern auch die Arbeit an einem Kapitel zur Methodologie der Psychosomatik in seiner *Methodenlehre*, wenngleich er dieses nicht abschließend realisieren konnte. Veränderungen werden schließlich auch im Selbstverständnis Martinis als klinischem Wissenschaftler erkennbar. „Subjektives“ und „Geistig-Seelisches“ findet sich wohl auch in früheren Arbeiten Martinis, hatte aber eher den Charakter von Lippenbekenntnissen. Nach 1949 setzte bei ihm eine tiefgründige Reflexion über deren Relevanz und Bedeutung in der klinischen Forschungsmethodik ein, „weil nichts Ärztliches sein kann, was nichts Subjektives an sich hat“ (Martini 1949b: 1352).

Wenn Martini solcherart die Bedeutung der Subjektivität in klinischer Forschung und ärztlicher Praxis anerkannte und zu differenzieren wusste, so wollte er diese jedoch nicht in einen Gegensatz zum „Zuverlässigkeitsanspruch“ der Naturwissenschaften geraten lassen (Martini 1949a). Dies zeigt sich insbesondere in der Frage der Kausalität: Für Martini war Kausalität ein kategoriales und leitmotivisches Forschungsprinzip, das sich mit klinisch-experimentellen und probabilistischen Begründungszusammenhängen plastisch machen ließ. Konkrete Kriterien für das Erkennen und Gewichten kausaler Zusammenhänge in der klinischen Forschung entwickelte er jedoch nicht. Die dafür maßgeblichen Impulse kamen in den 1960er Jahren aus der britischen Epidemiologie (Russo & Williamson 2007). Weizsäcker und Mitscherlich wiederum hielten den Kausalitätsbegriff in der Psychosomatischen Medizin für unanwendbar und letztlich obsolet, da man in einem als egalitär und reziprok aufgefassten Beziehungsgeschehen von Soma und Psyche nicht wissen könne, „wer angefangen hat“ (Weizsäcker 1952: 159).

Differenzen zeigen sich nicht zuletzt in der Frage, was in der klinischen Medizin unter Evidenz verstanden und anerkannt werden kann. Weizsäckers Evidenzverständnis war konsequent subjektzentriert und bezog sich auf Erkenntnis von im Patienten verborgenen seelisch-körperlichen „Umgangsformen“ (Weizsäcker 1949b: 17). Im Entbergen dieser Umgangsformen sah Weizsäcker gleichzeitig den Anspruch auf Erneuerung der Heilkunde realisiert. Mitscherlichs Plädoyer, Krankheit in ihrer Menschlichkeit aufzusuchen und mit ihren „Ausdrucksbewegungen“ augenscheinlich zu machen, weist in eine ähnliche Richtung. Der von Martini ausdrücklich und wiederholt gebrauchte Evidenzbegriff war anders gelagert. Er zielte auf die Erkenntnis kausaler Zusammenhänge im Krankheitsverlauf und ihrer nachgewiesen wirksamen Beeinflussung („klinischer Beweis“). Auch die Idee einer abgestuften, graduierten Evidenz findet sich bei ihm. Martinis Evidenzverständnis wies solcherart spezifische Komponenten auf, die er epistemologisch exponierte – und in die Reflexion klinischer Wissenschaftlichkeit integrierte. Allerdings verband er mit dem Evidenzbegriff keine programmatischen Ambitionen oder paradigmatischen Ansprüche, wie sie Jahrzehnte später die EbM-Bewegung setzte (Raspe 2018, Borck 2020). Der Versuchung, in Martini einen direkten Vorläufer der Evidenzbasierten Medizin zu sehen, wäre damit zu widerstehen.

### Archivbestände

Archiv der DFG, Bonn: Forschungsanträge und Karteikarten August W. v. Eiff, Paul Martini

Bundesarchiv Koblenz: B 136/921, B 136/916

Institute for Medical Humanities, Universität Bonn: Nachlass Paul Martini, Nr. 47 (Arbeiten und Korrespondenz zur Psychosomatik/Psychoanalyse), Nr. 77 (Kongress der Deutschen Gesellschaft für Innere Medizin)

Universitätsbibliothek Frankfurt am Main, Archivzentrum: Na 7 – Nachlass Alexander Mitscherlich (Korrespondenz Mitscherlich mit Curt Oehme: Oehme an AM, 16.05. und 29.05.1946; AM an Oehme, 03.05.1947)

## Footnotes

1 Anonymus 1949

2 Korrespondenz Paul Martini mit Curt Oehme 1948/49, Nachlass Martini. Vier Wochen nach Abhaltung des Kongresses vom 25.–28. April 1949 wurde das Grundgesetz für die Bundesrepublik Deutschland verkündet.

3 Bezeichnend ist hier ein von Bergmann an Martini gerichteter Brief vom 11. März 1948, in dem dieser sein Missfallen an Martinis Reorganisationsbemühungen zum Ausdruck bringt, da „man mit dem grossen Kongress noch ein Jahr Zeit gehabt hätte, bis die leidigen Qualifizierungen der Spruchkammern ausgestanden sind und die einzelnen Kollegen sich wieder ganz unbefangen benehmen“ (NL Martini, Nr. 77).

4 Auf die Bedeutung des Kongresses für die Geschichte der Psychosomatischen Medizin haben jüngst Geisthövel & Hitzer (2019: 194) hingewiesen.

5 Auch die anderen Beiträge dieser Special Section wählen den Kongress als Fokus und Bühne, um in exemplarischer Weise unterschiedliche Auffassungen von wissenschaftlicher Evidenz in der klinischen Medizin herauszuarbeiten. Vgl. hierzu die Einleitung der Gastherausgeber sowie den Beitrag von Roelcke zu Thure von Uexküll, Dörre zu Alexander Mitscherlich sowie von Rotzoll zu Jürg Zutt.

6 „Was sagen Sie dazu, dass die Mediziner in solchem Umfang der unverschämten Schwindelei der ‚Psychosomatischen Medizin‘ auf den Leim gehen? Auf dem Internistenkongress in Wiesbaden scheint – nach Bericht der ‚Psyche‘ – nur Ihr Bonner Martini wirklich vernünftig gesprochen zu haben.“ Jaspers an Hans Walter Gruhle, 10.01.1950, Nachlass Jaspers, DLA Marbach; vgl. Jaspers (1950).

7 Dehli (2007: 159).

8 Unmittelbar vor seinem Auftritt in Wiesbaden schrieb Mitscherlich an Arthur Jores, Martinis Damaskus-Formulierung ironisch wendend: „Dass die klinischen Oberhäupter in Wiesbaden kein Saulus-Paulus-Erlebnis haben werden, darüber bin ich mir völlig klar“ (Hoyer 2008: 184).

9 Zu Mitscherlichs weiterem Weg sowie zur Institutionalisierung der Psychosomatik in Heidelberg und der Psychoanalyse in Frankfurt (Sigmund-Freud-Institut) vgl. Hoyer (2008) und Roelcke (2004).

10 Thomä habilitierte sich zum Thema *Anorexia nervosa: Geschichte, Klinik und Theorien der Pubertätsmagersucht*. Bern: Huber (1961).

11 Eiff verlagerte in der Folge sein Forschungsinteresse auf Stressreaktionen und wurde 1973 in Bonn auf eine internistische Professur mit Schwerpunkt Psychosomatik und vegetatives Nervensystem berufen. Vgl. Hofer (2014: 400).

12 Auch der zu diesem Zeitpunkt noch in Berlin wirkende Horst-Eberhard Richter erinnerte in einem Vortrag über *Methodenprobleme der psychosomatischen Medizin *mit Bezug auf Martini daran, dass aufseiten der (psychoanalytischen) Psychosomatik das Interesse an seelischen Konflikten nicht zu einer Vernachlässigung von Untersuchungen im somatischen Bereich führen dürfe (Richter 1961: 245).

13 Erste Entwürfe zu der 1964 von Albert Görres herausgegebenen *Denkschrift zur Lage der ärztlichen Psychotherapie und psychosomatischen Medizin* kommentierte Martini mit neugierigem Interesse, war an der weiteren Ausarbeitung jedoch nicht beteiligt: Paul Martini an Gerhard Hess (1955–1964 Präsident der DFG), 01.06.1960, NL Martini.

14 Brocher war von 1958 bis 1963 geschäftsführender Vorsitzender der DGPT (jeweils unter dem Vorsitz von Mitscherlich), vgl. https://dgpt.de/ueber-uns/geschichte-der-dgpt/vorstandsmitglieder-2021-1945 (05.09.2021).

15 Ebd. Martinis Einschätzung ist vor dem Hintergrund seiner Teilnahme an der internationalen Konferenz zur Bewertung und Weiterentwicklung der *Controlled Clinical Trials *zu sehen. Die Konferenz wurde 1959 in Wien unter dem Vorsitz von Austin Bradford Hill und mit Unterstützung des Council for International Organizations of Medical Sciences (CIOMS) abgehalten. Vgl. Bird (2014).
